# Gray Matter Alterations in Early and Late Relapsing-Remitting Multiple Sclerosis Evaluated with Synthetic Quantitative Magnetic Resonance Imaging

**DOI:** 10.1038/s41598-019-44615-3

**Published:** 2019-05-31

**Authors:** Christina Andica, Akifumi Hagiwara, Koji Kamagata, Kazumasa Yokoyama, Keigo Shimoji, Asami Saito, Yuki Takenaka, Misaki Nakazawa, Masaaki Hori, Julien Cohen-Adad, Mariko Yoshida Takemura, Nobutaka Hattori, Shigeki Aoki

**Affiliations:** 1 Department of Radiology, Juntendo University Graduate School of Medicine, Tokyo, Japan; 2 Department of Radiology, The University of Tokyo Graduate School of Medicine, Tokyo, Japan; 3 Department of Neurology, Juntendo University School of Medicine, Tokyo, Japan; 4 Department of Radiology, Tokyo Metropolitan Geriatric Hospital and Institute of Gerontology, Tokyo, Japan; 5 Department of Radiological Sciences, Graduate School of Human Health Sciences, Tokyo Metropolitan University, Tokyo, Japan; 6 Institute of Biomedical Engineering, Polytechnique Montreal, Montreal, Quebec Canada; 7 Functional Neuroimaging Unit, CRIUGM, Université de Montréal, Montreal, Quebec Canada

**Keywords:** Diagnostic markers, Multiple sclerosis

## Abstract

Extensive gray matter (GM) involvement has been demonstrated in multiple sclerosis (MS) patients. This study was aimed to identify GM alterations in relapsing-remitting MS (RRMS) patients using synthetic quantitative MRI (qMRI). We assessed myelin volume fraction (MVF) in each voxel on the basis of R1 and R2 relaxation rates and proton density in 14 early and 28 late (disease duration ≤5 and >5 years, respectively) RRMS patients, and 15 healthy controls (HCs). The MVF and myelin volumes of GM (GM-MyVol) were compared between groups using GM-based spatial statistics (GBSS) and the Kruskal-Wallis test, respectively. Correlations between MVF or GM-MyVol and disease duration or expanded disability status scale were also evaluated. RRMS patients showed a lower MVF than HCs, predominantly in the limbic and para-limbic areas, with more extensive areas noted in late RRMS patients. Late-RRMS patients had the smallest GM-MyVol (20.44 mL; early RRMS, 22.77 mL; HCs, 23.36 mL). Furthermore, the GM-MyVol in the RRMS group was inversely correlated with disease duration (r = −0.43, *p* = 0.005). In conclusion, the MVF and MyVol obtained by synthetic qMRI can be used to evaluate GM differences in RRMS patients.

## Introduction

Although multiple sclerosis (MS) is mainly known as a chronic inflammatory and demyelinating white matter (WM) disease, it also exhibits extensive gray matter (GM) involvement^1^. GM changes have been shown to occur in the earliest stages of the disease across different clinical subtypes, to be progressive over time, and to be related to physical disabilities and cognitive impairment in MS patients^2,3^. The pathological hallmark of the GM damage in MS is demyelination, which is frequently accompanied by axonal loss and microglial activation^4^.

MRI plays a key role in the diagnosis and monitoring of disease activity and response to therapy in MS. However, T2-weighted imaging (WI) and fluid-attenuated inversion recovery (FLAIR) imaging are poorly sensitive for visualization of GM lesions^3,5–7^. Double inversion recovery (DIR) and phase-sensitive inversion recovery (PSIR) imaging can show greater sensitivity and specificity for GM lesions compared to FLAIR imaging due to enhanced tissue contrast^6^. However, these MRI techniques do not allow the quantification of tissue damage in the normal-appearing GM areas^6^.

Quantitative MRI (qMRI) provides an absolute measurement of the physical parameters of the underlying tissue microstructure and hence enables a more objective evaluation of disease^8^. In MS, qMRI allows evaluation of the development and progression of GM pathology by, for example, indirect mapping of myelin density^6^. Various qMRI techniques have been widely used to assess the development and progression of GM pathology in MS, such as magnetization transfer (MT) imaging and myelin imaging based on proton density (PD) and transverse relaxation times of myelin water^6,9^. However, MT imaging depends not only on various imaging parameters, necessitating correction and normalization^5^, but it is also influenced by edema and inflammation^10^. Single-component analysis of R1, R2, and PD is somewhat insensitive in showing MS-related demyelination in WM because these parameters can also be influenced by other pathologies^11,12^. For more direct imaging of myelin, one method was developed based on the measurement of the multi-exponential transverse T2 relaxation time. The shorter T2 component was attributed to water trapped between the myelin bilayers, termed myelin water, and the longer component was attributed to the combination of intra-/extra-axonal water. The term myelin water fraction (MWF) was defined as the ratio of myelin water to total water^13^. The correlation between MWF with the myelin content has been verified histopathologically in MS^14,15^. More recently, fast macromolecular proton fraction (MPF)  mapping^16^ and combined T2* and T1 cortical mapping (combined myelin estimation map)^17^ have also been used to evaluate the GM in MS. Although these methods show more specificity for myelin, their clinical use is hindered by their long acquisition times.

The synthetic qMRI method, with a multidynamic multiecho sequence, enables simultaneous quantification of R1 and R2 relaxation rates and PD, with B1 field inhomogeneity correction, in a single acquisition within a clinically acceptable time, and has demonstrated accuracy and reproducibility, even across different vendors^18–20^. The acquired data can be analyzed using software called ‘SyMRI’, with a post-processing time of less than 1 minute; synthetic contrast-weighted imaging, such as T1-WI, T2-WI, FLAIR, short-T1 inversion recovery (STIR), DIR, and PSIR imaging, automatic brain segmentation, such as WM, GM, and CSF, and myelin measurements, can also be performed after the acquisition without any additional scanning^18,21,22^. Based on the quantitative R1, R2, and PD values, a 4-compartment method was developed to estimate myelin volume fraction (MVF) in the brain^23^. In short, the myelin model is based on the assumption that each acquisition voxel is composed of four partial volumes—myelin, cellular, free water, and excess parenchymal water—and has its own R1, R2, and PD. MVF in a voxel is then calculated based on the effect of myelin on intra- and extracellular water relaxation rates due to magnetization exchange^23^. One of the major advantages of this approach is that the synthetic myelin map can be used to directly estimate the volume fraction of myelin in a voxel; thus, this method does not require a scaling factor. A more detailed description of this method is discussed in the Materials and Methods section. The model has shown good repeatability^24^ and correlation with myelin-sensitive Luxol fast-blue stained post-mortem brain assessments^25^ and other myelin imaging techniques^26^. It can also quantify demyelination of WM in MS^27,28^. The MVF, R1, R2, and PD values of the plaques, periplaque WM, and normal-appearing WM were shown to be different, and MVF showed higher sensitivity compared to the other metrics^27,28^. Additionally, in the healthy pediatric population, whole brain myelin volume (MyVol) measured by synthetic qMRI was well-fitted to the maturation curve^29^.

We hypothesized that MVF obtained by synthetic qMRI offers increased sensitivity for the detection of GM changes in the brain of early and late relapsing-remitting MS (RRMS) patients compared to other synthetic qMRI metrics, such as R1, R2, and PD and cortical thickness and subcortical volume. To test this hypothesis, we obtained GM-based spatial statistics (GBSS) to compare MVF, R1, R2, and PD between healthy controls (HCs) and early and late RRMS patients. We also compared GM-myelin volumes (GM-MyVol) and assessed intergroup differences in the cortical thickness and subcortical volume. Finally, we investigated the correlation between MRI measurements and clinical scores, such as disease duration and Expanded Disability Severity Scale (EDSS) scores.

## Results

### Study participants

Table 1 summarizes the demographic and clinical characteristics of HCs and RRMS patients. No significant difference was observed with regard to age (*p* = 0.20) and sex (*p* = 0.07) between the HCs and early and late RRMS groups. As expected, the late RRMS group had a significantly longer disease duration compared to the early RRMS group (*p* = 0.00002). The early RRMS did not differ from the late RRMS groups with regard to EDSS scores (*p* = 0.30). None of the patients showed extensive signal changes or more than two plaques in the GM.

**Table 1:** Demographic characteristics of the participants.

	HC	Early RRMS	Late RRMS	*p*-value	Post-hoc^‡^		
					HC vs. Early	HC vs. Late	Early vs. Late
Number	15	14	28				
Age, mean (SD) (years)*	41.07 (9.19)	36.07 (8.27)	41.18 (7.66)	0.20			
Sex (male/female)^†^	2/13	6/8	4/24	0.07			
Disease duration, mean (SD) (years)^‡^	NA	3.29 (1.54)	12.14 (6.59)	0.00002			
EDSS score, median (range)^‡^	NA	0.5 (0–2.5)	1 (0–6.5)	0.30			
GM-MyVol, mean (SD) (mL)^¶^	23.67 (4.12)	22.90 (2.37)	19.80 (2.96)	0.0006	0.90	0.002	0.002
Whole GM-Vol, mean (SD) (mL)^¶^	694.71 (48.03)	725.48 (78.54)	675.44 (53.78)	0.10			

GM-Vol, gray matter volume; GM-MyVol, myelin volume of gray matter; HC, healthy control; NA, not applicable; RRMS, relapsing-remitting multiple sclerosis; SD, standard deviation. Note: the statistical analyses were performed with one-way ANOVA*, χ^2^ test^†^, non-parametric Kruskal Wallis^¶^, and Mann-Whitney U test^‡^.

### GBSS analysis

**Figure 1:**
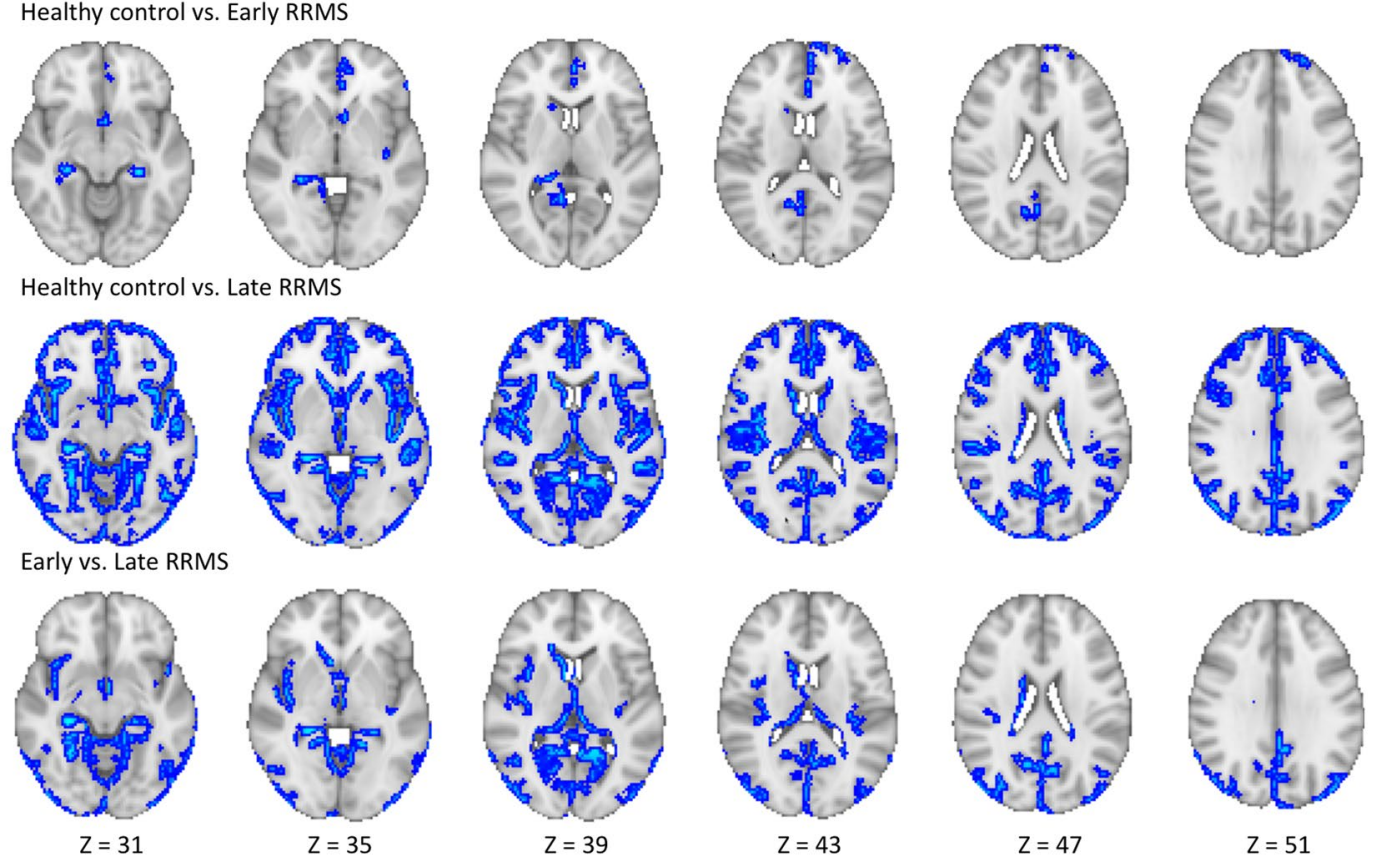
Comparison of myelin volume fraction (MVF) obtained from synthetic quantitative MRI between healthy controls and early and late relapsing-remitting multiple sclerosis (RRMS) patients. Gray-matter based spatial statistical analysis showed significantly lower MVFs (blue-light blue voxels) in early and late RRMS patients when compared with healthy controls and in late RRMS patients when compared with early RRMS patients. To aid visualization, the results (family-wise error corrected *p* < 0.05) are thickened using the fill script implemented in the FMRIB software library.

RRMS patients had a lower MVF; the differences were more pronounced in those with late RRMS than in those with early RRMS and HCs. We observed that the significant regions were not distributed evenly; therefore, we have reported the results in order from the largest to the smallest number of significant voxels. Early RRMS patients had lower MVFs than HCs in the limbic and para-limbic, frontal, parietal, occipital, and deep GM areas (Fig. 1, Table 2). Late RRMS patients had lower MVFs than HCs in the limbic and para-limbic, frontal, temporal, parietal, occipital, and deep GM areas and in the limbic and para-limbic, temporal, occipital, parietal, deep GM, and frontal areas when compared with those with early RRMS (Fig. 1, Table 2).

**Table 2:** GBSS analysis of MVF in early- and late-RRMS patients compared to healthy controls and in late RRMS compared to early RRMS.

Significant areas		Number of significant voxels	Peak *p*-FWE	Peak MNI Coordinates		
				X	Y	Z
Healthy control vs. Early RRMS			0.03	29	44	33
Frontal	Lt-Medial Orbito Frontal; Lt-Superior Frontal	21				
Parietal	Rt-Pre-cuneus	15				
Occipital	Rt-Lingual	2				
Limbic and Para-limbic	Lt-Caudal Anterior, Rostral Anterior Cingulate; Rt-Isthmus Cingulate; Lt-Insula; Lt-Rt-Hippocampus	103				
Deep GM	Rt-Thalamus, Rt-Caudate	5				
Healthy control vs. Late RRMS			0.0002	29	59	12
Frontal	Lt-Rt-Caudal Middle, Lateral Orbito, Medial Orbito, Rostral Middle, Superior Frontal; Lt-Rt-Frontal Pole; Lt-Rt-Paracentral; Lt-Rt-Pars Opercularis; Lt-Rt-Pars Orbitalis; Lt-Rt-Pars Triangularis; Lt-Rt-Precentral	1013				
Temporal	Lt-Rt-Bankssts; Lt-Rt-Entorhinal; Lt-Rt-Fusiform; Lt-Rt-Inferior, Middle, Superior, Transverse Temporal; Lt-Rt-Temporal Pole	974				
Parietal	Lt-Rt-Inferior Parietal; Lt-Superior Parietal; Lt-Rt-Post Central; Lt-Rt-Precuneus; Lt-Rt-Supramarginal	423				
Occipital	Lt-Cuneus; Lt-Rt-Lateral Occipital; Lt-Rt-Lingual; Lt-Rt-Pericalcarine	471				
Limbic and Para-limbic	Lt-Rt-Isthmus, Posterior, Rostral Anterior cingulate; Rt-Caudal Anterior Cingulate; Lt-Rt-Parahippocampal; Lt-Rt-Hippocampus; Lt-Rt-Insula; Lt-Rt-Amygdala; Lt-Rt-Accumbens	1499				
Deep GM	Lt-Rt-Thalamus, Lt-Rt-Caudate, Lt-Rt-Putamen	189				
Early vs. Late RRMS			0.003	33	40	26
Frontal	Lt-Paracentral; Rt-Precentral; Lt-Superior Frontal; Rt-Lateral Orbito Frontal	24				
Temporal	Lt-Rt-Entorhinal; Lt-Rt-Fusiform; Lt-Rt-Inferior, Middle, Transverse Temporal; Lt-Superior temporal; Lt-Rt-Temporal Pole;	333				
Parietal	Lt-Rt-Inferior Parietal; Rt-Superior Parietal; Lt-Rt-Precuneus; Lt-Rt-Supramarginal	139				
Occipital	Lt-Cuneus; Lt-Rt-Lateral Occipital; Lt-Rt-Lingual; Lt-Rt-Pericalcarine	143				
Limbic and Para-limbic	Lt-Rt-Isthmus Cingulate; Lt-Posterior Cingulate; Lt-Rt-Parahippocampal; Lt-Rt-Hippocampus; Lt-Rt-Insula; Lt-Rt-Amygdala; Rt-Accumbens	695				
Deep GM	Lt-Rt-Thalamus; Lt-Putamen; Rt-Caudate	90				

Lt, left; Rt, right; Bankssts, banks of superior temporal sulcus; GM, gray matter; GBSS, gray-matter–based spatial statistics; *p*-FWE, family-wise error-corrected *p*-value; MVF, myelin volume fraction; RRMS, relapsing-remitting multiple sclerosis. Note: Only regions with a significantly decreased MVF are included.

Late RRMS patients had a significantly higher PD than HCs in the limbic and para-limbic, temporal, frontal, occipital, and deep GM areas (Supplementary Fig. 1, Supplementary Table 1). Further, they had a significantly lower R2 than HCs in the limbic and para-limbic, temporal, frontal, parietal, deep GM, and occipital areas and in the limbic and para-limbic, temporal, parietal, and frontal areas when compared with those with early RRMS (Supplementary Fig. 2, Supplementary Table 2). In early RRMS patients, no significant differences were found in PD in comparison with HCs and late RRMS patients; in R2 when compared with HCs; and in R1 for all group comparisons. There were no significant correlations between MVF, R1, R2, or PD and disease duration or EDSS scores in RRMS patients.

### ROI analysis of MVF, R1, R2, and PD

ROI analysis showed significantly lower MVFs in the frontal, temporal, parietal, occipital, limbic and para-limbic, and deep GM areas of RRMS patients in all group comparisons. Differences were more pronounced in late RRMS patients than in those with early RRMS and HCs. A significantly lower R2 was found in the frontal, temporal, parietal, occipital, limbic and para-limbic, and deep GM areas of late RRMS patients than in HCs and early RRMS patients. Although there were significant changes in R2 in the frontal areas of early-RRMS patients than in HCs, the results were inconsistent. A description of the significant areas can be seen in Supplementary Tables 3 and 4 for MVF and R2, respectively. No statistically significant differences were found in R1 and PD in all group comparisons. There were no significant correlations between MVF, R1, R2, or PD and disease duration or EDSS scores in RRMS patients.

### Whole GM and GM-myelin volumetry

**Figure 2:**
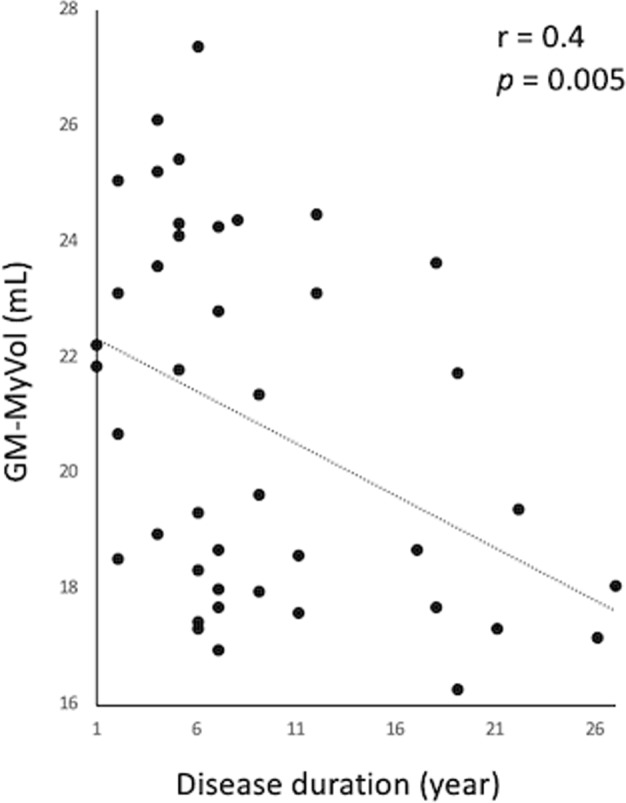
Scatterplots showing a negative correlation between gray matter myelin volume (GM-MyVol) and disease duration in relapsing-remitting multiple sclerosis patients.

No significant intergroup differences were found in whole GM-Vol (Table 1). Late RRMS patients showed significantly lower GM-MyVol [19.8 (standard deviation (SD) 2.93) mL] than HCs (23.67 (SD 4.12) mL, *p* = 0.002) and those with early RRMS (22.9 (SD 2.37) mL, *p* = 0.002). Furthermore, GM-MyVol in the total RRMS group was inversely correlated with disease duration (r = −0.43. *p* = 0.005) (Fig. 2).

### Cortical thickness and subcortical volume analysis

No significant differences were detected in cortical thickness in all group comparisons. Early RRMS patients had a significantly smaller pallidum (*p* = 0.02) than HCs. Late RRMS patients had a smaller thalamus (*p* = 0.0008), putamen (*p* = 0.002), and pallidum (*p* = 0.0005) than HCs and a significantly smaller thalamus (*p* = 0.009) and putamen (*p* = 0.004) than those with early RRMS (Table 3). There were no significant correlations between cortical thickness or subcortical volume and disease duration or EDSS scores in RRMS patients. No significant correlation was found between whole cortical thickness and MVF.

**Table 3:** Subcortical volume analysis in early and late RRMS patients compared to healthy controls and in late RRMS compared to early RRMS.

	HCs Mean (SD) in mm^3^	Early RRMS Mean (SD) in mm^3^	Late RRMS Mean (SD) in mm^3^	FDR corrected-*p*	Post-Hoc		
					HCs vs. Early RRMS	HCs vs. Late RRMS	Early vs. Late RRMS
Deep GM							
*Left*							
Thalamus	8.2 (0.7)	7.9 (1.1)	7.0 (1.2)	0.04	NS	0.0008	0.009
*Right*							
Putamen	5.2 (0.8)	5.0 (0.9)	4.2 (0.8)	0.04	NS	0.002	0.004
Pallidum	1.5 (0.1)	1.3 (0.2)	1.2 (0.3)	0.04	0.02	0.0005	NS

Note: Only regions with FDR-corrected *p* < 0.05 are shown.

## Discussion

In this study, we used synthetic qMRI to evaluate the GM changes in the brains of early- and late-stage RRMS patients. A lower MVF, which reflects demyelination, was found in RRMS patients when compared with HCs and in late RRMS patients when compared with those exhibiting early RRMS. These findings are in agreement with previous histopathological and radiological studies showing that demyelination is a major pathology finding in the GM of MS patients, and GM changes can be seen from an early stage of the disease^4,30^. Our study also demonstrated that the lower MVF in late-RRMS patients involved more extensive areas than those in early RRMS patients, suggesting that the GM pathology may progress over time, as was shown in previous longitudinal studies^2,31^.

In line with previous studies^6,32–34^, our study also showed that the GM damage in RRMS patients is not evenly distributed. In the GBSS analysis, a lower MVF was predominantly seen in the limbic and para-limbic areas in both early- and late-RRMS patients. However, the orders of other affected areas were slightly different in these groups, with the frontal and temporal cortices being the second most affected in early- and late-RRMS patients, respectively. As reported by previous histopathological^33^, MT imaging^34^, and cortical thickness and volume evaluation^32^ studies, the limbic system seems to be more susceptible than other GM regions in the early stage of MS. Additionally, the frontal and temporal lobes have been shown to be affected slightly more than the parietal or occipital lobes^6^. Although the exact origin of GM changes in MS is debatable, subpial demyelination extending across multiple gyri (“general subpial demyelination”) has been shown to be the cause of the observed pattern of cortical changes^35^. Meanwhile, axonal damage in WM lesions might lead indirectly to anterograde and retrograde degeneration of axons running within the deep GM and contribute to demyelination by reducing local metabolic activity^1^.

We also demonstrated that a voxel-wise GM analysis with GBSS enables the regional mapping and characterization of GM pathology in RRMS patients. Generally, the PVE of the WM and CSF adjacent to the cortical GM produces some difficulties in obtaining accurate measurements of the cortical GM, a thin structure (a few mm) with complicated convolutional patterns. In the voxel-based analysis (VBA), a common method of automated exploratory analysis, smoothing is generally performed to reduce individual differences, and this procedure further increases the PVE of the WM and CSF^36^. The GBSS method used in this study was developed based on the modification of the VBA for cortical analyses, by aggregating the parameters of regions surrounding a skeleton created at the center of the cortical GM, thereby minimizing the PVE of the WM and CSF^37^.

RRMS patients showed a smaller GM-MyVol than HCs, with the late-RRMS group showing the lowest value. Our study is the first to evaluate the total myelin volume of GM. Although different tissue-class probability thresholds can have profound effects on segmentation results and lead to different values for volume calculation^38^, the GM-MyVol data acquired in this study might be used as reference or baseline values for follow-up by using a constant threshold. This is reinforced by the fact that GM-MyVol was inversely correlated with disease duration.

Increased PD and prolonged T1 and T2 (the inverse of R1 and R2) have been related to demyelination^11,12^. In the present study, GBSS analysis showed a significantly higher PD and lower R2 only in the late-RRMS patients, with no significant difference in R1. In other words, these metrics were not sensitive enough to show the GM damage in the early stages of RRMS. Our results are in line with a previous study showing that MVF obtained by synthetic qMRI is more sensitive than R1, R2, and PD values in showing abnormalities in MS plaques and periplaque WM^27^. This may be partly because changes in R1, R2, and PD can also be caused by other pathological conditions. PD can be affected by any changes in tissue water content, such as edema; R1 and R2 can be reduced because of changes in axonal density and gliosis^11,12^; and R2 can show a strong increase with abnormal iron deposition^11^.

Assessment of GM atrophy has been widely performed in the MS population, with inconsistent results. While some studies have shown evidence of GM atrophy in the early stage of MS^32^, others have not^39^. The present study showed significant deep GM volume loss in early- and late-RRMS patients, with a higher degree of loss in late RRMS. However, the cortical thickness was not different between the three groups. A decrease in MVF was seen more extensively than GM atrophy in RRMS patients in this study, which is in line with a previous study showing that a decrease in MTR (also reflecting demyelination) was more extensive than atrophy^40^. One possible explanation for this is that GM atrophy is mainly caused by axonal loss rather than demyelination, which has been shown in a histopathological study^41^. This is further supported by the finding of this study, as there was no significant correlation between cortical thickness and MVF.

None of the metrics in this study were correlated with EDSS scores. EDSS scores are a measure of disability known to be primarily sensitive to ambulation in MS and are not a sensitive assessment of worsening disease in MS patients, thus, obscuring disability progression in patients with higher levels of disability^42^. This observation is in line with the findings of previous studies that showed no correlation between EDSS scores and PD, MTR, and T2 in the GM of RRMS patients. In contrast, Yarnykh *et al.*^16^ revealed significant correlations between MPF and EDSS. However, they evaluated the whole GM of not only RRMS patients, but also secondary-progressive MS patients with a wide range of EDSS scores, which might have led to this discrepancy.

It is important to reiterate that synthetic qMRI myelin measurement is not a direct method. Theoretically, the sequence is not fast enough to catch the rapid relaxation of myelin water. Instead, MVF is calculated by its effect of magnetization exchanges between intra- and extracellular water and the decrease in observed PD^26^. The assumptions regarding constant rates of relaxation and exchange for the four partial volume compartments are indeed a limitation of the model. Furthermore, even though a synthetic myelin model considers the magnetization exchange rate between tissue compartments, it does not explicitly integrate a partial volume pool to account for magnetization transfer effects^23,43^. The magnetization transfer effect may result in small changes of Carr-Purcell-Meiboom-Gill multiecho signal amplitudes and propagate to the R2 estimations. However, our myelin estimation model is estimated from combinations of the measured R1, R2, and PD values, and the effect of any potential offset in R2 is expected to be small^28^. Iron accumulation has been demonstrated in MS as the effect of iron release, which is stored primarily within oligodendrocytes and myelin fibers upon demyelination^44^. An increase in iron predominantly increases the R2^45^. If the R2 rate exceeds the predefined grid of MVF, as shown in the study by Warntjes *et al*.^23^, the measured R1, R2, and PD coordinates will be projected onto the point that is nearest that is included in the grid, which will still provide an estimate of myelin. In that sense, the presence of excessive iron concentrations or deposits are not accounted for in the myelin model, which could lead to local estimation errors. This method might have some limitations compared with more direct myelin measures, such as the  MWF, which estimates the distribution of T2 of myelin water by using a multi-exponential T2 decay^46^. Nevertheless, our study shows that the MVF and GM-MyVol derived from synthetic qMRI can be useful for the evaluation of MS patients. Further, a previous study have shown that the synthetic myelin map has the highest contrast between WM and GM compared to other myelin imaging techniques, matched with the histopathological studies^26^. Finally, synthetic qMRI has some advantages in the clinical setting, such as good repeatability of MVF assessments^24^ and the ability to create a wide range of contrast-weighted images, such as T1-WI, T2-WI, FLAIR, STIR, DIR, and PSIR, by post-processing without additional scans^18^.

Some other limitations of this study should be addressed. First, this study only included a small sample size. The small sample size limits the power of the statistical analyses and may have led to false positive or negative findings. Second, we only evaluated RRMS patients, and studies with different MS phenotypes are needed to validate the robustness of this method. Third, cognitive assessment was not performed and evaluated in this study. A previous study showed that GM damage, especially in the hippocampus and deep GM, has a strong correlation with cognitive impairment in MS^47^. Further research should be performed to evaluate the correlation between synthetic qMRI parameters with cognitive impairment in MS. Fourth, the relatively thin cortex and the 4-mm slice thickness on synthetic 2D qMRI acquisition might have caused measurement errors in the metrics related to a relative increase in the number of partial volume voxels at the CSF/brain tissue interface^48^. However, in this study, GBBS was used to minimize the partial volume contamination by skeletonizing the GM and projecting the metrics to a cortical ribbon^37^. Finally, due to the limitation of the MR scanner, we used 3D spoiled gradient recalled echo (SPGR) T1-WI in the evaluation of cortical thickness, while 3D magnetization-prepared rapid gradient-echo (MPRAGE) T1-WI is preferred in the FreeSurfer pipeline because of the good contrast between GM, WM, and CSF^49^.

In summary, our study demonstrated that synthetic MVF offers greater sensitivity than other metrics, such as R1, R2, PD, and cortical thickness and subcortical volume, in the detection of GM abnormalities in RRMS patients. GBSS analysis showed that a lower MVF was predominantly seen in the limbic and para-limbic areas followed by other GM areas of early- and late-RRMS patients. This study also showed lower GM-MyVol values in RRMS patients, which were inversely correlated with disease duration. Taken together, this study supports the notion that MVF and GM-MyVol obtained by synthetic qMRI might be useful in the evaluation of MS patients. However, considering the limitations of this study, the findings should be clinically interpreted with caution.

## Materials and Methods

### Study participants

All data from the participants were obtained following the 2013 revised Helsinki Declaration of 1964. This study was approved by the institutional review board of Juntendo University Hospital. Written informed consent was obtained from each participant after we provided detailed information.

Forty-two RRMS patients and 15 age- and sex-matched HCs with no history of neurological or psychiatric diseases were prospectively included in the study from May 2016 to November 2016. As defined by Chard *et al*.^30^, RRMS was categorized as early (≤5 years) or late RRMS (>5 years) based on the disease duration. Neurological disability in these patients was assessed by EDSS. The demographic and clinical characteristics of all participants are shown in Table 1.

### MRI acquisition and post-processing

All participants underwent synthetic 2D axial qMRI and 3D SPGR T1-WI on a 3 T MR scanner (Discovery MR750w; GE Healthcare, Milwaukee, Wisconsin, USA) with a 19-channel head coil. Synthetic qMRI was performed using a MAGiC (magnetic resonance imaging compilation) pulse sequence—a multi-slice, multi-echo, and multi-saturation delay saturation-recovery turbo spin-echo acquisition method. Combinations of two echo times (TEs, 16.9 and 84.5 ms) and four delay times (146, 546, 1879, and 3879 ms) were used to generate eight complex images that were then used to quantify R1, R2, and PD. We utilized parallel imaging with an acceleration factor of 2. Furthermore, acquired data were reconstructed by zero interpolation filling to a matrix of 512 × 512, which was automatically performed on the MR scanner using a default setting. The other acquisition parameters of synthetic qMRI were as follows: repetition time (TR), 4 s; field of view (FOV), 240 × 240 mm; matrix size, 320 × 320, echo-train length (ETL), 10; slice thickness/gap, 4.0 mm/1.0 mm; acquisition time, 7′ 12″. The acquisition parameters of 3D T1WI were: TE/TR, 3.09 ms/7.7 ms; FOV, 256 × 256 mm; matrix size, 256 × 256; ETL, 1, slice thickness/gap, 1.0 mm/-, respectively.

After image acquisition, SyMRI software version 8.0 (SyntheticMR, Linköping, Sweden) was used to acquire myelin, R1, R2, and PD maps (to obtain MVF, R1, R2, and PD values, respectively), synthetic contrast-weighted images, GM mask, and whole GM volume (GM-Vol) with 2D synthetic qMRI data. Then, an experienced neuroradiologist (CA), who was blinded to the clinical information, used synthetic FLAIR (TR, 15000 ms; TE, 100 ms; TI, 3000 ms), synthetic DIR (TR, 15000 ms; TE, 100 ms; TI/TI2, 470/3750 ms), and synthetic PSIR (TR, 6000 ms; TE, 10 ms; TI, 500 ms) images to evaluate the GM lesions.

### Myelin measurements

Warntjes *et al*.^23^ proposed a model in which each acquisition voxel is assumed to be composed of four partial volumes: myelin partial volume; cellular partial volume; free water partial volume; and excess parenchymal water partial volume, where each partial volume has its own R1, R2, and PD^23^. The estimation of myelin partial volume in this model considers magnetization exchange between the myelin water component trapped between the myelin sheaths and the surrounding intracellular and extracellular water^18,23^. This was performed by running Bloch equations and optimizing model parameters in a spatially normalized and averaged brain from a group of 20 healthy controls^23^.

The synthetic qMRI approach measures the B1+ field and assumes that the B1- field is identical, which becomes an increasing deviation at higher field strengths. However, phantom measurements show that the effect on the final calculated PD is generally small (<5%)^19^. The B1+/B1− issue does not affect the measurement of R1 and R2. All three parameters, R1, R2, and PD, are used for the calculation of myelin and therefore an error of 5% would be the upper limit. In addition, the myelin measurement is not performed using the amplitude of multiexponential decay parameters, which would be mathematically impossible with only two echoes. Instead, it measures the dominant, slow-decay component of both T1 and T2 relaxation (and the amplitude of PD) to infer the presence of myelin. It is therefore the effect of the magnetization exchange of the fast myelin water with the observed cellular water that is detected, and not the fast component itself. We consider the two-echo approach reliable to measure T2 relaxation as it is corrected for RF pulse profile effects and B1 inhomogeneity effects and shows high accuracy in phantom measurements in comparison to a high number of echoes^20^.

### GBSS analysis

GBSS^37^ was used to compare MVF, R1, R2, and PD between groups and to perform correlation analyses between MVF, R1, R2, or PD and disease duration or EDSS scores. GBSS is an analogue pipeline of tract-based spatial statistics (TBSS)^50^ used to perform voxel-wise statistical analysis on GM and ensures accurate anatomical alignment between subjects by using a skeleton projection step^37^. The partial volume effect (PVE) of WM and CSF can lead to errors in the evaluation of GM. By creating a skeleton and consolidating parameter of the surrounding regions into that skeleton, it is possible to avoid PVE of the WM and CSF. Furthermore, in the presence of pathology associated with regional morphological changes, classic registration methods can present difficulty in normalizing an individual’s brain anatomy to a common template. Herein, we used GBSS to reduce the effects of residual spatial misalignment between subjects that may have resulted from the complex cortical geometry and pathology.

Image processing was performed using the FMRIB Software Library version 5.0.9 (FSL)^51^. First, the Brain Extraction Tool (BET) was used to remove non-brain tissue from each subject’s 3D T1-WI. Then, each skull-stripped 3D T1-WI was affine- and nonlinearly aligned to an MNI152 standard space at a 2-mm resolution using FMRIB’s linear image registration tool (FLIRT) and FMRIB’s nonlinear image registration tool (FNIRT)^52^, respectively. In the pipeline, the full Jacobian was internally calculated and used. Further, field bias was corrected and GM, WM, CSF segmentations were obtained with FMRIB’s automated segmentation tool (FAST)^53^. The resulting GM image was used to create a mean GM skeleton with a threshold of 0.2 to minimize the contribution of voxels from WM and CSF^54^. Finally, the synthetic myelin, R1, R2, and PD maps of each subject were registered to their corresponding 3D T1-WI using FLIRT and were aligned to standard space by using the registration matrix and warping field created for 3D T1-WI^52^.

### Region of interest (ROI) analysis

We measured the mean MVF, R1, R2, and PD values of the GM skeleton of each subject of 82 ROIs, obtained from the Desikan-Killiany atlas, which includes 34 cortical and seven subcortical parcellated regions on both sides.

### GM-myelin volume (GM-MyVol) analysis

Although SyMRI software provides automatic whole-brain MyVol calculation, this study focused on GM. Therefore, we extracted GM-MyVol by using FSL. First, we obtained synthetic GM masks from the SyMRI software. Then, we thresholded the mask at 0.90^19^ to avoid the PVE of WM and CSF. As a considerable portion of deep GM was segmented as WM using the SyMRI software, we further created a deep GM mask using FSL. The MNI152 standard space was registered to each patient’s synthetic T1-WI using FLIRT and FNIRT. Then, the FMRIB Integrated Registration and Segmentation Tool (FIRST)^55^ was applied to create a deep GM mask. Finally, the total GM mask was created by adding the thresholded synthetic GM and deep GM masks and then applied to the myelin map. The total GM-MyVol was calculated by multiplying the summed MVFs and the volume of voxels.

### Cortical thickness and subcortical volume measurements

Cortical thickness and subcortical volume measurements were performed with FreeSurfer pipeline (http://surfer.nmr.mgh.harvard.edu/fswiki) as previously described^56^ by using 3D SPGR T1-WI. The cortical and subcortical areas then were labeled with the Desikan-Killiany atlas (82 ROIs in total).

### Statistical analysis

All statistical analyses were performed using IBM SPSS Statistics for Windows, Version 22.0 (IBM Corporation, Armonk, NY, USA), except for the general linear model (GLM) analyses, which were performed using FSL. The Shapiro-Wilk test was used to assess the normality of the data. The demographic and clinical data were analyzed with the Mann-Whitney U test (2 groups) and one-way analysis of variance (ANOVA) (3 groups) for continuous variables and the χ^2^ test for categorical variables. The threshold of statistical significance for all analyses was set at *p* < 0.05.

For GBSS, a voxel-wise GLM framework with one-way ANOVA, was applied to compare MVF, R1, R2, and PD between groups by using the FSL’s *randomise* tool with 5,000 permutations. The results were then corrected for multiple comparisons by controlling family-wise error (FWE) and applying threshold-free cluster enhancement (TFCE). The same method was applied to perform a voxel-wise correlation analysis between MVF, R1, R2, and PD of all RRMS patients with disease duration or EDSS scores.

The mean values of MVF, R1, R2, PD, cortical thickness, and subcortical volume were compared between groups with nonparametric Kruskal-Wallis tests. Then, the Benjamini-Hochberg false discovery rate (FDR) correction was applied to correct multiple testing (82 ROIs). Pairwise comparisons were then performed to detect significant main effects on any area with significant FDR-corrected *p*-values by conducting nonparametric Mann-Whitney U tests. Group differences in cortical thickness were also assessed using a GLM with one-way ANOVA and corrected for multiple comparisons by Monte Carlo simulation on the significant clusters using 10,000 permutations.

Total GM-Vol and GM-MyVol differences between groups were assessed with nonparametric Kruskal-Wallis tests. Pairwise comparisons for significant main effects were performed with the Mann-Whitney U test.

The relationship between the mean values of MVF, R1, R2, PD, GM-MyVol, or cortical thickness and subcortical volume in the whole RRMS group and disease duration or EDSS scores was assessed with Spearman’s correlation coefficient. FDR correction was applied only for multiple testing (82 ROIs) of MVF, R1, R2, PD, and cortical thickness and subcortical volume.

Finally, the relationship between the mean values of the whole cortical MVF and cortical thickness in the whole group were also assessed with Spearman’s correlation coefficient.

## Supplementary information

Supplementary dataset 1

## Data Availability

The datasets generated during and/or analyzed during the current study are available from the corresponding author on reasonable request.
