# Supplementary material for: Gray Matter Alterations in Early and Late Relapsing-Remitting Multiple Sclerosis Evaluated with Synthetic Quantitative Magnetic Resonance Imaging
Source: Sci Rep. 2019 May 31;9:8147. doi: 10.1038/s41598-019-44615-3 (PMC6544650; doi:10.1038/s41598-019-44615-3)
Supplement: Supplementary file 1 — Supplementary dataset 1 [file 41598_2019_44615_MOESM1_ESM.pdf]

Gray Matter Alterations in Early and Late Relapsing-Remitting Multiple Sclerosis Evaluated with Synthetic Quantitative Magnetic Resonance Imaging

Christina Andica[1], Akifumi Hagiwara[1,2*], Koji Kamagata[1], Kazumasa Yokoyama[3], Keigo Shimoji[illegible], Asami Saito[1], Yuki Takenaka[1,5], Misaki Nakazawa[1], Masaaki Hori[1], Julien Cohen-Adad[6,7], Mariko Yoshida Takemura[1], Nobutaka Hattori[3], Shigeki Aoki[3]

[1]Department of Radiology, Juntendo University Graduate School of Medicine, Tokyo, Japan

[2]Department of Radiology, The University of Tokyo Graduate School of Medicine, Tokyo, Japan

[3]Department of Neurology, Juntendo University School of Medicine, Tokyo, Japan

[4]Department of Radiology, Tokyo Metropolitan Geriatric Hospital and Institute of Gerontology, Tokyo, Japan

[5]Department of Radiological Sciences, Graduate School of Human Health Sciences, Tokyo Metropolitan University, Tokyo, Japan

[6]Institute of Biomedical Engineering, Polytechnique Montreal, Montreal, Quebec, Canada

[7]Functional Neuroimaging Unit, CRIUGM, Université de Montréal, Montreal, Quebec, Canada

Supplementary Table 1. GBSS analysis of PD in late RRMS patients compared to healthy controls.

Significant areas		Number of significant voxels	Peak *p*-FWE	Peak MNI Coordinates		
				X	Y	Z
Healthy control vs. Late RRMS			0.05	37	47	31
Frontal	Rt-Latero Orbito Frontal	32				
Temporal	Rt-Entorhinal; Rt-Fusiform; Rt-Temporal pole	72				
Occipital	Lt-Rt-Lingual; Lt-Rt-Pericalcarine	38				
Limbic and Para-limbic	Lt-Posterior Cingulate; Rt-Isthmus Cingulate; Rt-Hippocampus; Rt-Amygdala; Rt-Parahippocampal; Rt-Insula	872				
Deep GM	Rt-Thalamus, Rt-Putamen	144				

Lt, left; Rt, right; GM, gray matter; GBSS, gray-matter–based spatial statistics; *p*-FWE, family-wise error-corrected *p*-value; PD, proton density; RRMS, relapsing-remitting multiple sclerosis. Note: Only regions with significantly decreased PD are included.

Supplementary Table 2. GBSS analysis of R2 late RRMS patients compared to healthy controls and early RRMS patients.

Significant areas		Number of significant voxels	Peak *p*-FWE	Peak MNI Coordinates		
				X	Y	Z
Healthy control vs. Late RRMS			0.01	53	33	41
Frontal	Lt-Lateral Orbito, Medial Orbito, Rostral Middle, Superior Frontal; Lt-Pars Opercularis; Lt-Pars Orbitalis; Lt-Pars Triangularis; Lt-Frontal pole; Lt-Precentral	168				
Temporal	Lt-Rt-Entorhinal; Lt-Rt-Fusiform; Lt- Middle, Superior, Transverse Temporal; Lt-Rt-Inferior Temporal; Lt-Temporal Pole	235				
Parietal	Lt-Post Central; Lt-Rt-Precuneus; Lt-Supramarginal	123				
Occipital	Lt-Cuneus; Lt-Lingual; Lt-Pericalcarine	44				
Limbic and Para-limbic	Lt-Rt-Isthmus Cingulate; Lt-Rostral Anterior Cingulate; Lt-Rt-Parahippocampal; Lt-Rt-Hippocampus; Lt-Insula; Lt-Rt-Amygdala; Lt-Rt-Accumbens	575				
Deep GM	Lt-Rt-Thalamus; Lt-Rt-Caudate; Lt-Putamen	91				

Early vs. Late RRMS			0.02	66	56	31
Frontal	Lt-Pars Opercularis; Lt-Precentral	16				
Temporal	Lt-Superior, Transverse Temporal	40				
Parietal	Lt-Post Central; Lt-Supramarginal	26				
Limbic and Para-limbic	Lt-Insula	69				

Lt, left; Rt, right; GM, gray matter; GBSS, gray-matter–based spatial statistics; *p*-FWE, family-wise error-corrected *p*-value RRMS, relapsing-remitting multiple sclerosis. Note: Only regions with significantly decreased R2 are included.

Supplementary Table 3. ROI analysis of MVF in early and late RRMS patients compared to healthy controls and in late RRMS compared to early RRMS.

	HC<br>Mean (SD)	Early RRMS<br>Mean (SD)	Late RRMS<br>Mean (SD)	FDR<br>corrected-*p*	Post-Hoc Analyses<br>HCs vs. Early RRMS	 HCs vs. Late RRMS	 Early vs. Late RRMS
Frontal							
Left							
Caudal Middle Frontal	9.42 (1.95)	8.72 (1.27)	8.15 (1.45)	0.08	NS	0.04	NS
Lateral Orbito Frontal	11.83 (0.88)	10.91 (0.75)	10.42 (1.40)	0.002	0.01	0.0003	NS
Rostral Middle Frontal	9.87 (0.74)	9.36 (1.19)	8.88 (1.36)	0.03	NS	0.007	NS
Superior Frontal	10.62 (1.49)	9.85 (1.20)	9.37 (1.13)	0.02	NS	0.005	NS
Frontal Pole	11.80 (1.71)	10.22 (2.17)	10.16 (2.25)	0.04	NS	0.009	NS
Paracentral	9.78 (0.97)	9.51 (0.85)	8.63 (2.11)	0.05	NS	0.03	0.05
Precentral	11.77 (0.83)	10.75 (0.97)	10.39 (1.10)	0.003	0.008	0.0004	NS
Right							
Medial Orbito Frontal	11.32 (0.93)	10.06 (1.31)	9.69 (1.35)	0.001	0.03	0.00003	NS

Lateral Orbito Frontal	11.63 (0.95)	10.44 (1.58)	10.09 (1.45)	0.005	NS	0.0006	NS
Superior Frontal	10.77 (1.13)	9.81 (0.93)	9.17 (1.54)	0.001	0.02	0.0002	0.02
Precentral	11.62 (1.05)	10.47 (0.81)	9.95 (1.32)	0.001	0.01	0.0002	NS
Pars Opercularis	10.03 (1.54)	8.84 (1.81)	8.51 (1.75)	0.02	NS	0.004	NS
Pars Orbitalis	8.39 (1.48)	7.47 (0.85)	6.81 (1.51)	0.009	NS	0.003	NS
Pars Triangularis	11.56 (1.63)	11.10 (1.10)	10.00 (2.00)	0.01	NS	0.01	0.02
Temporal							
Left							
Banks of the superior temporal sulcus	14.55 (1.50)	13.99 (1.49)	12.95 (2.14)	0.03	NS	0.01	NS
Entorhinal	6.15 (0.86)	5.99 (0.98)	5.25 (1.33)	0.006	NS	0.003	0.01
Fusiform	9.02 (1.16)	8.13 (1.09)	7.61 (1.36)	0.007	0.04	0.001	NS
Inferior Temporal	8.63 (0.79)	7.91 (0.98)	7.13 (1.11)	0.001	NS	0.00007	0.02
Middle Temporal	8.45 (1.14)	7.35 (1.11)	6.90 (1.16)	0.003	0.02	0.0004	NS
Superior Temporal	10.73 (0.93	9.83 (1.07)	9.08 (1.21)	0.0008	0.02	0.00008	0.01
Transverse Temporal	16.29 (1.63)	14.80 (1.92)	13.44 (1.58)	0.0005	0.04	0.00003	0.008

Temporal Pole	6.57 (1.18)	6.06 (1.34)	5.41 (1.02)	0.006	NS	0.001	0.03
Right							
Banks of the superior temporal sulcus	13.19 (1.44)	12.79 (2.06)	11.34 (1.61)	0.006	NS	0.001	0.04
Fusiform	10.50 (1.53)	9.53 (1.59)	8.53 (1.92)	0.009	NS	0.002	NS
Inferior Temporal	8.57 (1.22)	8.13 (1.16)	7.25 (1.14)	0.003	NS	0.0006	0.009
Superior Temporal	10.80 (1.12)	9.95 (0.90)	9.10 (1.33)	0.0008	0.04	0.00007	0.009
Transverse Temporal	15.61 (1.50)	14.39 (2.20)	13.30 (2.27)	0.002	NS	0.0001	0.03
Temporal Pole	5.27 (1.02)	5.88 (1.37)	4.87 (1.04)	0.009	0.05	NS	0.002
Parietal							
Left							
Inferior Parietal	8.00 (0.78)	7.43 (0.73)	6.95 (1.21)	0.01	NS	0.003	NS
Superior Parietal	9.06 (0.88)	8.13 (0.82)	7.80 (1.40)	0.007	0.01	0.002	NS
Postcentral	13.31 (1.33)	12.01 (1.10)	11.66 (1.23)	0.003	0.01	0.0005	NS
Precuneus	9.91 (1.19)	9.25 (1.29)	8.36 (1.80)	0.006	NS	0.001	0.05
Supramarginal	13.39 (1.51)	12.41 (1.75)	11.86 (1.60)	0.04	NS	0.008	NS

Right							
Inferior Parietal	8.82 (0.98)	7.96 (1.19)	7.61 (1.45)	0.02	NS	0.005	NS
Postcentral	11.53 (1.08)	10.88 (0.90)	10.10 (1.45)	0.006	NS	0.001	0.05
Precuneus	10.71 (1.14)	9.84 (1.45)	9.11 (1.73)	0.007	NS	0.001	NS
Supramarginal	14.08 (1.10)	12.03 (1.62)	11.88 (1.54)	0.001	0.001	0.00007	NS
Occipital							
Left							
Cuneus	6.09 (1.18)	6.05 (1.27)	4.96 (1.70)	0.04	NS	0.02	0.04
Lateral Occipital	7.12 (1.25)	6.92 (0.88)	5.97 (1.14)	0.004	NS	0.004	0.003
Lingual	9.31 (0.95)	8.97 (0.74)	7.90 (1.13)	0.0007	NS	0.0003	0.0004
Right							
Lateral Occipital	7.79 (0.77)	7.86 (0.67)	6.77 (1.03)	0.001	NS	0.002	0.0005
Lingual	11.08 (0.89)	10.33 (1.39)	9.45 (1.52)	0.003	NS	0.0004	0.03
Limbic and Para-Limbic							
Left							
Isthmus Cingulate	10.27 (1.69)	9.11 (1.25)	7.29 (2.12)	0.0009	NS	0.00009	0.008

Posterior Cingulate	11.01 (0.81)	9.62 (0.93)	8.60 (1.43)	0.001	0.0002	0.000004	0.01
Rostral Anterior Cingulate	10.26 (2.27)	8.76 (1.71)	7.85 (1.85)	0.009	NS	0.002	NS
Caudal Anterior Cingulate	8.80 (1.75)	7.59 (1.14)	7.30 (1.96)	0.03	NS	0.009	NS
Parahippocampal	10.07 (1.66)	8.86 (0.90)	8.03 (1.63)	0.003	NS	0.0004	0.03
Hippocampus	11.61 (1.38)	9.70 (1.70)	9.05 (2.09)	0.003	0.007	0.0005	NS
Insula	12.61 (1.31)	11.65 (1.66	10.99 (1.49)	0.007	NS	0.001	NS
Amygdala	9.68 (0.95)	9.18 (1.12)	8.64 (1.88)	0.07	NS	0.03	NS
Accumbens	10.28 (1.39)	8.71 (1.91)	8.72 (1.86)	0.02	0.02	0.005	NS
Right							
Isthmus Cingulate	10.62 (1.68)	9.32 (1.45)	8.24 (1.78)	0.002	NS	0.0003	0.03
Posterior Cingulate	11.69 (1.27)	10.28 (1.59)	9.45 (1.99)	0.003	0.03	0.0003	NS
Caudal Anterior Cingulate	8.87 (1.39)	7.77 (1.17)	7.24 (1.89)	0.009	NS	0.002	NS
Rostral Anterior Cingulate	11.35 (1.73)	9.87 (1.76)	8.57 (2.13)	0.001	0.03	0.0002	0.03
Parahippocampal	11.22 (1.54)	10.06 (1.29)	8.58 (1.72)	0.0005	NS	0.00004	0.004
Hippocampus	12.22 (1.48)	10.30 (1.56)	9.04 (2.02)	0.0005	0.01	0.00001	0.01
Insula	14.01 (1.21)	12.77 (1.81)	11.44 (1.71)	0.0005	NS	0.000008	0.02

Deep gray matter							
Left							
Thalamus	13.22 (0.80)	12.31 (1.59)	11.44 (1.81)	0.004	0.04	0.001	NS
Putamen	12.18 (0.88)	11.58 (1.18)	11.00 (1.44)	0.01	NS	0.003	NS
Right							
Thalamus	13.65 (1.10)	12.98 (1.30)	11.77 (1.68)	0.002	NS	0.0002	0.03
Caudate	16.50 (3.14)	13.39 (2.69)	13.23 (3.49)	0.01	0.009	0.007	NS
Putamen	11.88 (1.02)	11.40 (1.01)	10.91 (1.22)	0.03	NS	0.008	NS
Pallidum	15.03 (3.75)	13.97 (4.60)	12.00 (3.96)	0.04	NS	0.009	NS

FDR, false discovery rate; HC, healthy control; MVF, myelin volume fraction; NS, not significant; ROI, region of interest; RRMS, relapsing-remitting multiple sclerosis; SD, standard deviation. Note: Only regions with FDR-corrected $p < 0.05$ are shown.

Supplementary Table 4. ROI analysis of R2 in early and late RRMS patients compared to healthy controls and in late RRMS compared to early RRMS patients.

	HCs Mean (SD)	Early RRMS Mean (SD)	Late RRMS Mean (SD)	FDR corrected-*p*	Post-Hoc Analyses HCs vs. Early RRMS	HCs vs. Late RRMS	Early vs. Late RRMS
Frontal							
Left							
Medial Orbito Frontal	14.00 (0.94)	14.00 (0.78)	13.25 (0.84)	0.04	NS	0.01	0.008
Lateral Orbito Frontal	13.51 (0.56)	12.96 (0.59)	12.71 (0.70)	0.04	0.01	0.0006	NS
Rostral Middle Frontal	13.52 (1.27)	14.54 (1.35)	13.02 (1.45)	0.04	0.05	NS	0.004
Paracentral	14.24 (1.55)	14.47 (1.57)	13.15 (1.07)	0.04	NS	0.02	0.001
Pars Opercularis	14.00 (1.99)	14.61 (1.25)	12.30 (2.06)	0.04	NS	0.02	0.005
Precentral	13.87 (1.59)	15.37 (2.17)	13.13 (1.63)	0.04	NS	NS	0.002
Right							
Medial Orbito Frontal	14.16 (1.16)	14.03 (1.12)	13.13 (0.65)	0.04	NS	0.001	0.01
Temporal							

Left							
Inferior Temporal	13.83 (0.94)	14.02 (1.15)	13.07 (1.06)	0.04	NS	0.02	0.008
Middle Temporal	13.80 (1.03)	14.12 (1.10)	12.97 (0.89)	0.04	NS	0.005	0.004
Superior Temporal	14.19 (1.24)	15.65 (1.82)	13.99 (1.76)	0.04	NS	0.002	0.01
Transverse Temporal	14.80 (2.71)	16.08 (2.76)	13.40 (2.90)	0.04	NS	NS	0.003
Parietal							
Left							
Postcentral	14.62 (2.33)	16.17 (2.99)	13.80 (1.90)	0.04	NS	NS	0.005
Limbic and Para-limbic							
Left							
Posterior Cingulate	13.54 (1.18)	14.09 (1.10)	12.67 (1.04)	0.04	NS	NS	0.002
Insula	14.21 (1.27)	14.49 (1.58)	12.93 (1.73)	0.04	NS	0.01	0.006
Right							
Accumbens	12.85 (1.66)	13.67 (1.04)	12.25 (1.36)	0.04	NS	NS	0.001
Deep gray matter							
Left							

Putamen	13.60 (1.02)	13.21 (1.16)	12.20 (1.30)	0.04	NS	0.0004	0.04

FDR, false discovery rate; HC, healthy control; NS, not significant; ROI, region of interest; RRMS, relapsing-remitting multiple sclerosis; SD, standard deviation. Note: Only regions with FDR-corrected $p < 0.05$ are shown.

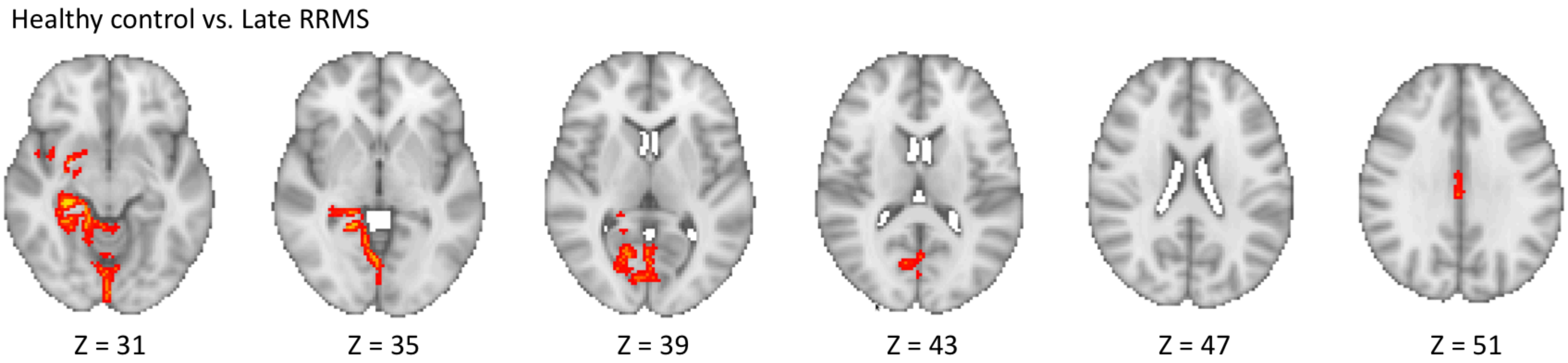

Supplementary Figure 1. Comparison of proton density (PD) determined using synthetic quantitative MRI between healthy controls and late relapsing-remitting multiple sclerosis (RRMS) patients. Gray-matter based spatial statistical analysis showed a significantly higher PD (red-yellow voxels) in late RRMS patients when compared with healthy controls. To aid visualization, the results (family-wise error corrected $p < 0.05$) are thickened using the fill script implemented in the FMRIB software library.

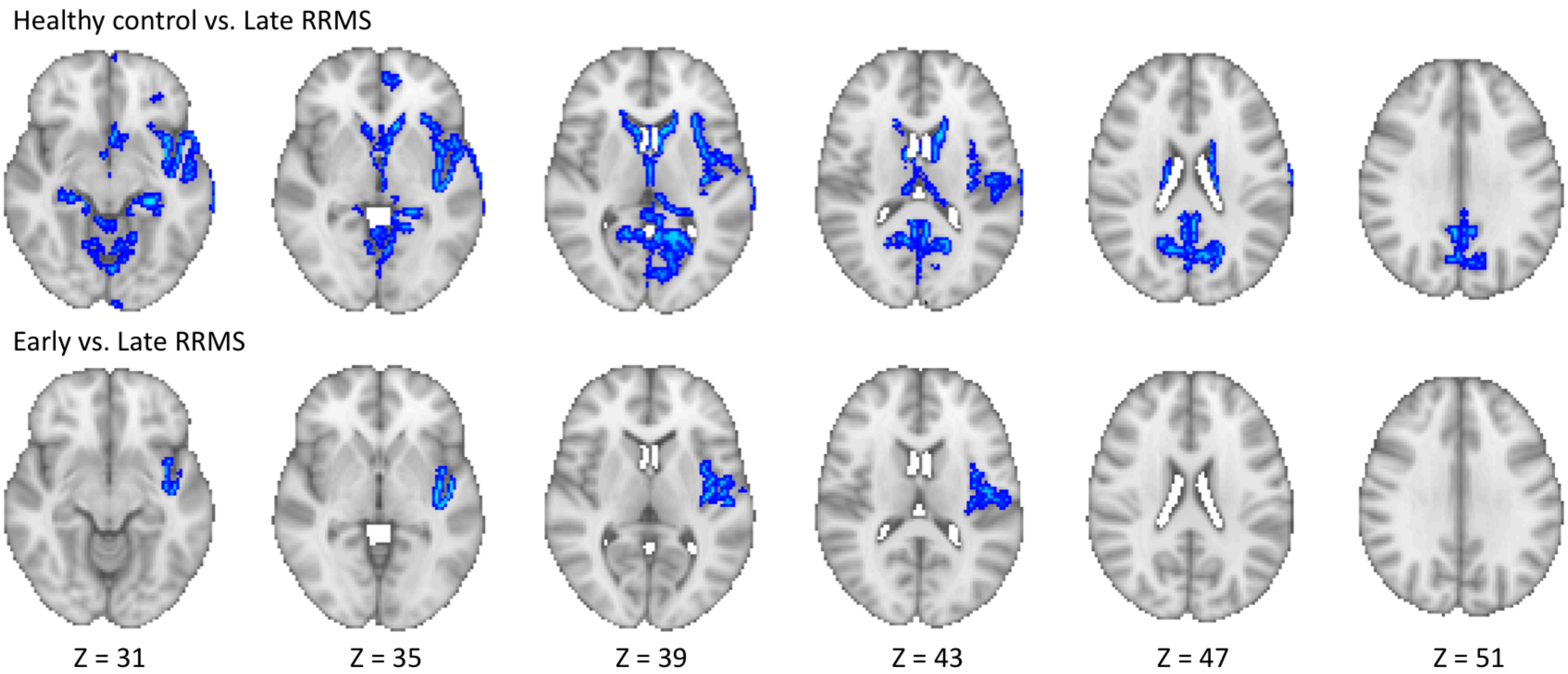

Supplementary Figure 2. Comparison of R2 values obtained from synthetic quantitative MRI between healthy controls and early and late relapsing-remitting multiple sclerosis (RRMS) patients. Gray-matter based spatial statistical analysis showed a significantly lower R2 (blue-light blue voxels) in late RRMS patients when compared with healthy controls and early RRMS patients. To aid visualization, the results (family-wise error corrected $p < 0.05$) are thickened using the fill script implemented in the FMRIB software library.
